# Editorial: The actions of trace element metabolism and epigenetics on animal health and disease

**DOI:** 10.3389/fvets.2022.1086322

**Published:** 2022-11-25

**Authors:** Demin Cai, Meijuan Tu, Dan Wan

**Affiliations:** ^1^Laboratory of Animal Physiology and Molecular Nutrition, College of Animal Science and Technology, Yangzhou University, Yangzhou, China; ^2^Department of Biochemistry and Molecular Medicine, University of California, Davis, Sacramento, CA, United States; ^3^Key Laboratory of Agro-ecological Processes in Subtropical Region, Institute of Subtropical Agriculture, Chinese Academy of Sciences, Changsha, China

**Keywords:** trace element, livestock, dietary intervention, minerals, anti-oxidation

## Introduction

Chronic exposure to toxic levels of trace elements might lead to gene expression dysfunction and abnormal metabolic pathways such as those linked to trace element detoxification and the reproductive performances of animals. It has been a subject consisting of a series of studies testing the physiological outputs when exposed to these trace elements and the underlying epigenetic modulation mechanisms such as DNA/RNA methylation, histone modifications, and microRNA dysregulation. Epigenetics plays a pivotal role in gene expression and is vulnerable to environmental challenges, including the supply of nutritional factors, such as vitamins and minerals. Minerals usually function as cofactors in the activation of epigenetically activating enzymes. As it is true for most epigenetically active factors, the mineral balance mostly determines the epigenome generation during embryonic development, but changes can be triggered throughout life as part of life-term epigenome editing. It has also been proved that alterations induced by minerals accumulation during aging could be passed on to the next generation. Together, mineral supplementation is suggested to prevent dysplasia originating from errors in establishing the epigenome or correct epigenetic disturbances to benefit animal health.

The aim of this Research Topic “*The actions of trace element metabolism and epigenetics on animal health and disease*” in Frontiers in Veterinary Science is to highlight and stimulate discussion regarding trace element accumulation-induced epigenetic modulation and toxicity/diseases (e.g., disruption of nutrients homeostasis and dysplasia) and the possible detoxication strategies. This topic has collected 4 scientific contributions from highly qualified research groups focusing on trace element metabolism in 4 different animal models including rabbit, pig, goat, and hen.

## The actions of trace element metabolism on animal health

Trace elements also known as coenzyme elements of several metal enzymes, exert pivotal actions in human and animal growth, metabolism, digestion, and absorption. Especially, zinc (Zn) and copper (Cu) are critical mineral elements for maintaining health, preventing diseases, and stimulating the growth and the immune system of livestock and poultry. Li et al. performed a series of examinations to access the consequences of dietary administration of diverting concentrations of Zn and Cu on anti-oxidant, mineral contamination and excretion, digestion ability, metal transport, and meat quality in finishing pigs. They demonstrated that Zn and Cu supplementation have no side effects on pigs growth performance or meat quality. Nevertheless, mineral contamination and excretion, the enzymes involved in digestion and anti-oxidation, as well as metal transporters were sensitized to these treatments at the finishing stage. These data would better reflect practical concerns on the safe utilization of mineral supplies.

Another interesting finding in hens from Chen et al. was that organic trace elements (Cu, Fe, Zn, and Mn) did not affect the growth performance but increased metal and zinc transporter similar to that of the aforementioned study. Moreover, the haugh unit, egg yolk weight, eggshell weight, and eggshell thickness were not changed. However, eggshell strength, illus height, and villus concealment ratio were remarkably improved together with the elevated serum Cu, Fe, Zn levels. It concluded that low levels of organic trace elements benefit late-laying hens' production performance (Chen et al.).

Given that Zn is one of the crucial redox inactive trace elements to perform the antioxidant roles by activating antioxidant enzymes and the stabilization of sulfhydryl groups, Kucková et al. evaluated the effects of Zn and thyme extract administration on the antioxidant capacities and mineral status in the blood and tissues of growing rabbits. In this model, the authors predicted that Zn and/or aromatic herb *T. vulgaris* supplementation would perform positive anti-oxidation actions in rabbits. The results exhibited that Zn administration significantly increased the glutathione peroxidase, total antioxidant, and thiol group levels in the kidney. Intriguingly, *T. vulgaris* and Zn supplementation significantly reduced the Zn concentration in the kidney and Cu content in the muscle, respectively. These suggested that the simultaneous supplementation of organic Zn and *T. vulgaris* improved the antioxidant response in rabbit kidneys to facilitate protection against oxidative stress.

Besides the organic mineral elements including Zn, Cu, Fe, and Mn, selenium (Se) also plays potential anti-oxidative roles in livestock *via* diminishing reactive oxygen species and lipid peroxidation. In this regard, Tian et al. tested the selenium-yeast (SY) on goats growth performance, meat quality, fatty acids, and amino acids profiles, along with the antioxidant activities in muscle at the growing stage. The data reflected that goats supplemented with SY did not impair growth performance, muscle chemical composition, pH values, and the loss of water, drip, and cooking. High-dose SY at 4.8 mg/kg supply improves the meat quality by enhancing dressing percentage, eye muscle area, and meat color associated with muscle total antioxidant enzyme activities. Furthermore, owing to the critical benefits of polyunsaturated fatty acids (PUFAs) for livestock health, the authors also provide the evidences that SY treatment markedly remodeled the fatty acid and amino acid profiles to strengthen the anti-oxidation protection accordingly.

## Conclusions

Exogenous trace element supplies are vital components of metalloproteins and cofactors of enzymic activities for many cellular processes. However, chronic exposure to high doses of trace elements may lead to epigenetic dysregulation and various kinds of diseases. Therefore, the development of new models and methods to monitor trace element absorption and disposition is highly needed. In addition, as an accumulation of trace elements is inevitable, investigation of the subsequent epigenetic modulation such as DNA and histone modification and microRNA regulation may help us reveal the mechanism of the toxicity and predict and prevent the toxicity effectively. Although the epigenetic regulations were not mentioned in these documents, these studies provided a better understanding of trace element metabolism and relative anti-oxidation functions in animals.

## Author contributions

DC wrote the introduction, conclusion, and the central part with comments on the cited papers and references. DC, DW, and MT contributed to the review and editing. All authors contributed to the article and approved the submitted version.

## Funding

This work was supported by the Natural Science Foundation of Jiangsu Province (BK20200932), Natural Science Foundation of the Higher Education Institutions of Jiangsu Province (20KJB230001), and the Priority Academic Program Development of Jiangsu Higher Education Institutions (PAPD).

## Conflict of interest

The authors declare that the research was conducted in the absence of any commercial or financial relationships that could be construed as a potential conflict of interest.

## Publisher's note

All claims expressed in this article are solely those of the authors and do not necessarily represent those of their affiliated organizations, or those of the publisher, the editors and the reviewers. Any product that may be evaluated in this article, or claim that may be made by its manufacturer, is not guaranteed or endorsed by the publisher.

